# Supplementary material for: Recurrent evolution of adhesive defence systems in amphibians by parallel shifts in gene expression
Source: Nat Commun. 2024 Jul 10;15:5612. doi: 10.1038/s41467-024-49917-3 (PMC11237159; doi:10.1038/s41467-024-49917-3)
Supplement: Supplementary file 1 — Supplementary Information [file 41467_2024_49917_MOESM1_ESM.pdf]

Supplementary Information

Recurrent evolution of adhesive defence systems in amphibians by parallel shifts in gene expression

Shabnam Zaman, Birgit Lengerer, Joris Van Lindt, Indra Saenen, Giorgio Russo, Laura Bossaer, Sebastien Carpentier, Peter Tompa, Patrick Flammang, Kim Roelants

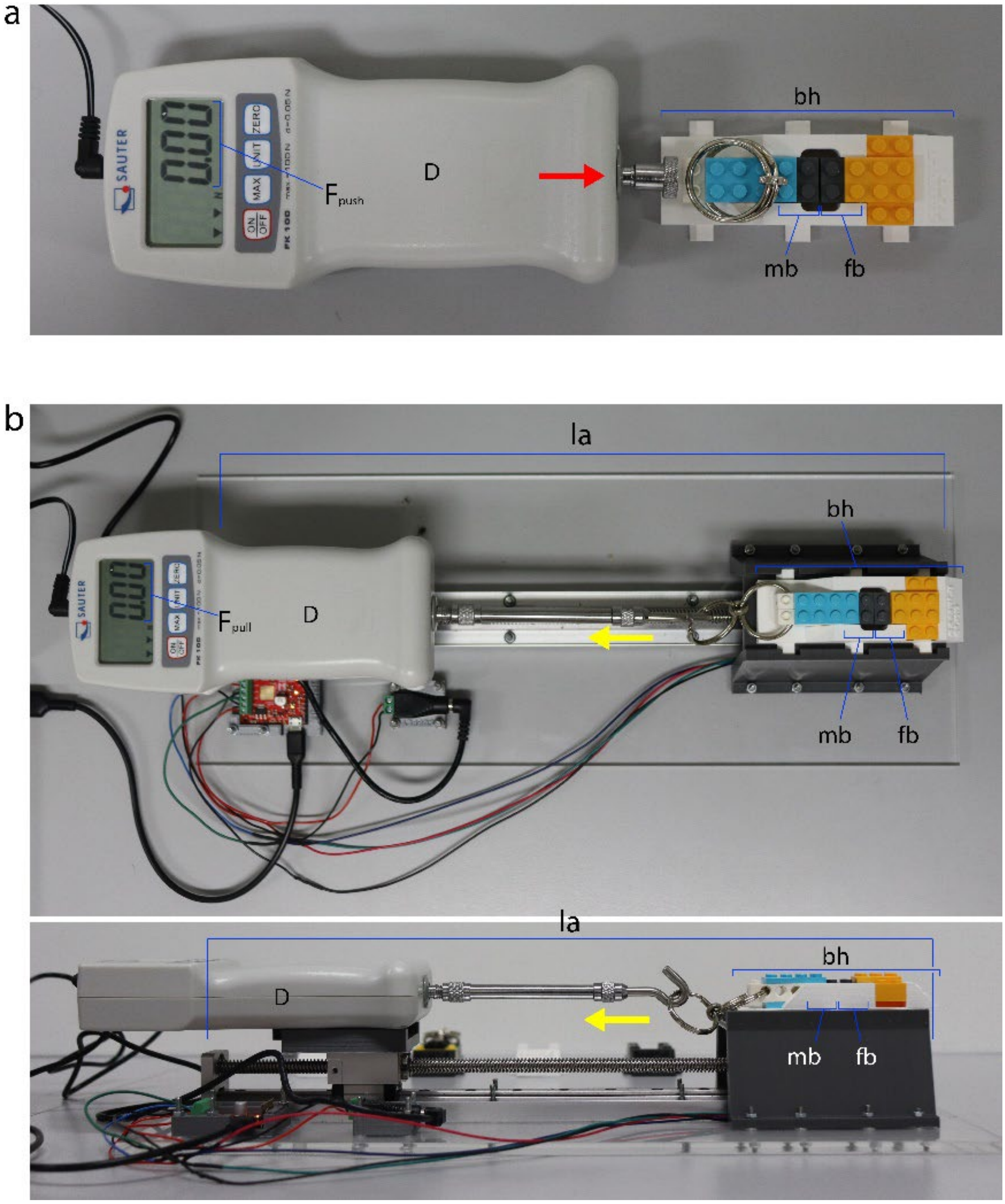

Supplementary Figure 1. Setup used for tensile strength measurements. **a** Bricks were glued together by slowly moving a dynamometer (*D*) forward (red arrow), pushing a movable brick (*mb*) coated with glue along a channel in a custom 3D-printed brick holder (*bh*) against a second brick (*fb*) fixed in the holder. Although the dynamometer was moved manually, it was pushed until reading a maximum push force (F_{push}; in N) that corresponded to one of two prespecified pressures (40 kPa or 4 kPa). **b** After curing for 10 or 60 minutes, the brick holder (*bh*) with glued bricks and dynamometer (*D*) were fixed on a motorised linear actuator (*la*) and the movable brick (*mb*) was hooked to the dynamometer. The actuator subsequently pulled the dynamometer away from the brick holder at a fixed velocity of 25 mm/min (yellow arrows), eventually detaching the movable brick from the fixed one (*fb*) along the holder's channel. The maximum pull force recorded before glue failure (F_{pull}; in N) was used to calculate the tensile strength (in kPa). *bh*, brick holder; *D*, dynamometer; *fb*, fixed brick; F_{pull}, pull force; F_{push}, push force; *la*, linear actuator; *mb*, movable brick.

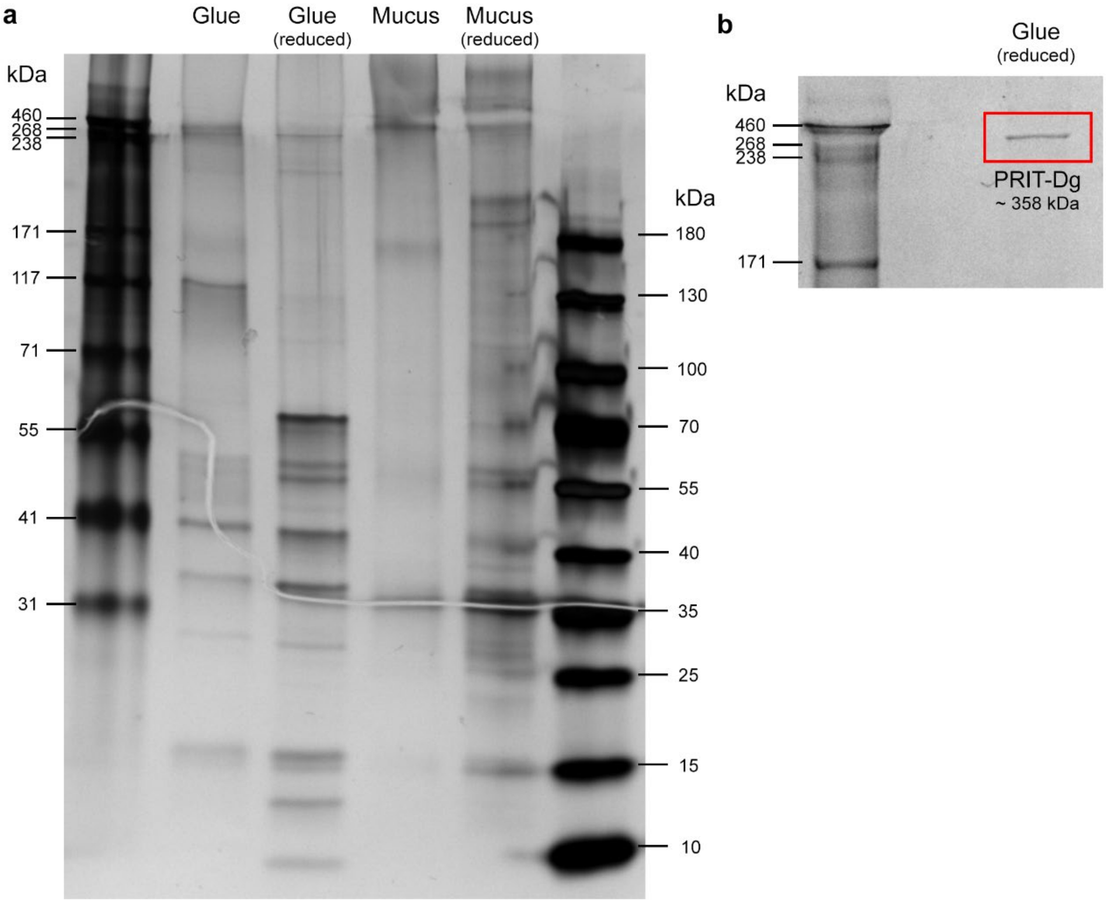

Supplementary Figure 2. SDS-PAGE gels after **a** silver staining to compare protein band profiles in two types of *Dyscophus guineti* secretion, glue and mucus, under nonreducing and reducing conditions, and **b** staining with Coomassie blue after an extended run of a reduced glue sample to obtain improved resolution of higher molecular weight bands. The indicated molecular weight estimate for PRIT-Dg is based on transcript sequencing (red box; see main text and Fig. 2b). Molecular weight band sizes are indicated in kDa.

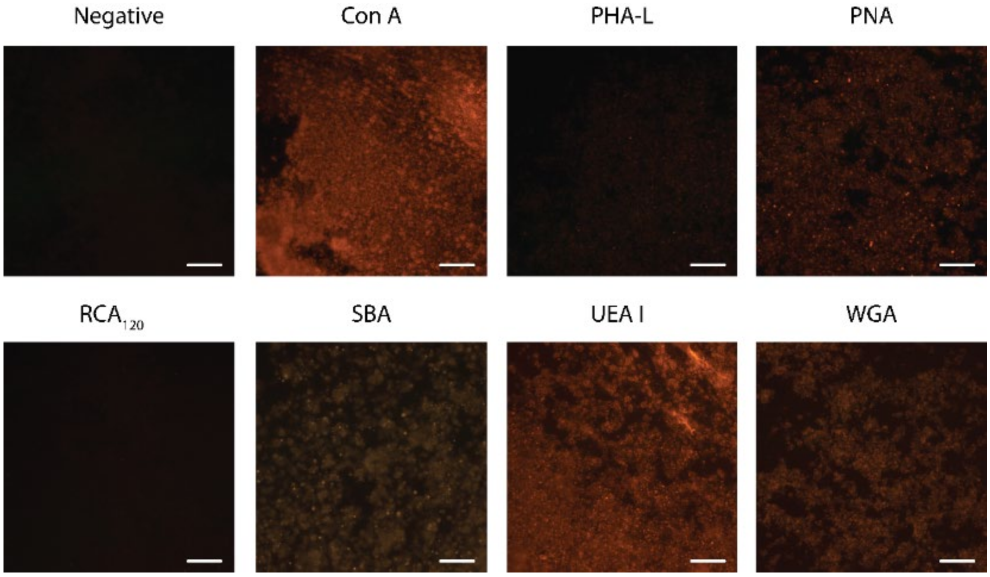

Supplementary Figure 3. Reactivity of *D. guineti* glue with lectins. The carbohydrate binding specificities of each lectin are as follows: Con A mannose, glucose; PHA-L galactose; PNA galactose; RCA_{120} galactose; SBA GalNac; UEA I fucose, arabinose; WGA GlcNAc. Scale bars: 50 µm.

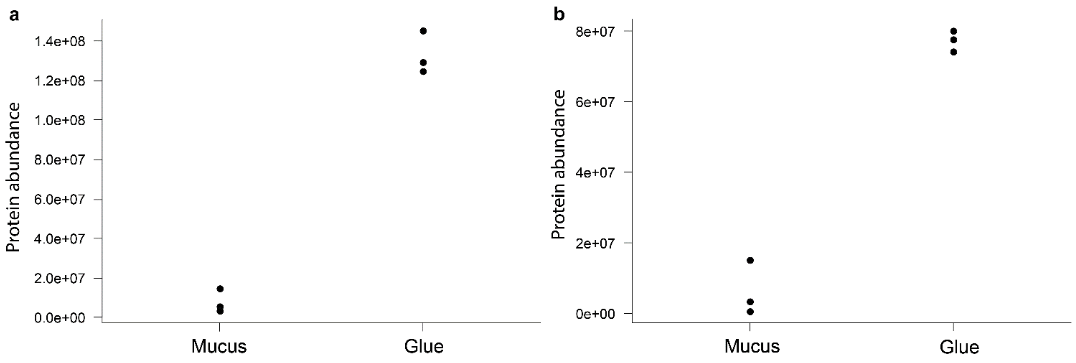

Supplementary Figure 4. Normalised protein abundances in two different secretion types, nonadhesive mucus and glue, estimated from mass spectrometry data for **a** PRIT-Dg and **b** galectin-Dg1 (n = 3 samples of each secretion type).

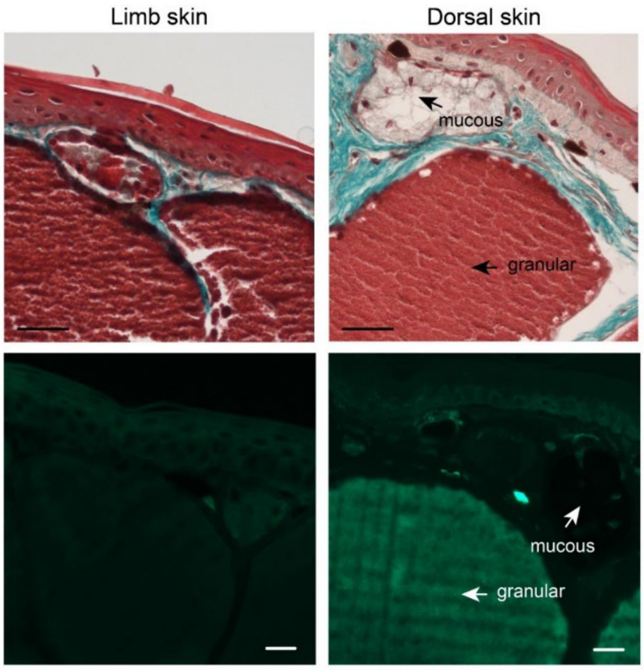

Supplementary Figure 5. Histological skin sections identify two different types of glands within *D. guineti* dorsal skin, mucous and granular (top). Immunohistochemical staining using anti-PRIT-Dg (interdomain repeats) as the primary antibody reveals that the PRIT-Dg protein is localised in dorsal granular glands, not mucous glands (bottom). Scale bars: 50 µm for histology sections (top), 20 µm for immunolabelled sections (bottom).

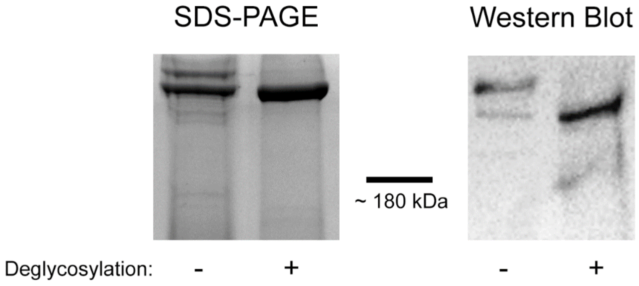

Supplementary Figure 6. Mobility shift of PRIT-Dg before (-) and after (+) treatment of *D. guineti* glue with enzymes to remove linked glycans associated with *N*- and *O*-glycosylation. For Western blotting, antibodies targeting the interdomain repeats of PRIT-Dg were used to detect changes in migration pattern. The size of the highest molecular weight marker in each gel is indicated in kDa.

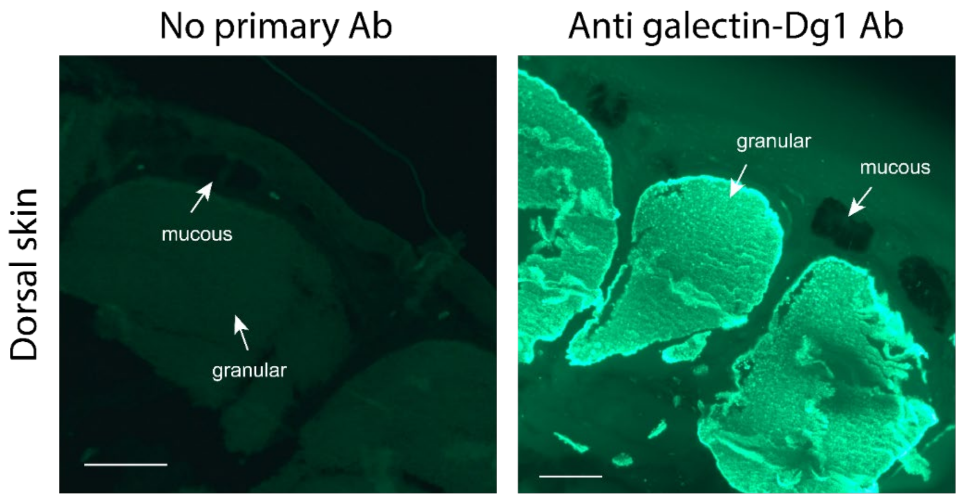

Supplementary Figure 7. Immunoreactivity assay of *D. guineti* dorsal skin with antibodies targeting galectin-Dg1 reveals that the protein is localised in dorsal granular glands, not mucous glands. Scale bars: 100 µm.

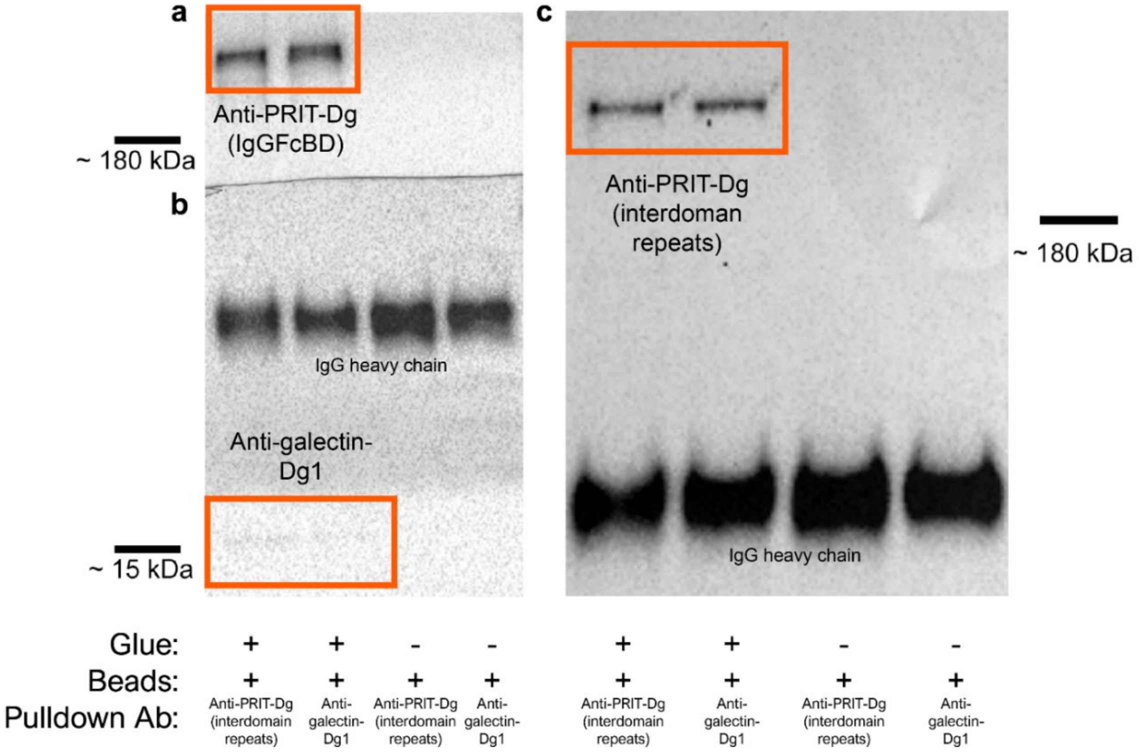

Supplementary Figure 8. Co-immunoprecipitation (co-IP) of glue proteins with antibodies directed against galectin-Dg1 confirms its interaction with PRIT-Dg as a binding partner on Western blots, and vice versa. Co-IP is used here to detect protein-protein interactions. In short, antibodies targeting one of the possible interacting proteins is coupled to beads and used to pull-down interacting protein complexes (pulldown Ab). The precipitate is then run on SDS-PAGE, followed by Western blotting with antibodies that specifically target possible interaction partners. This serves as a test to determine whether these partners do in fact co-precipitate together, thus forming a protein complex. Protein detection was carried out using antibodies specific to **a** the IgGFcBD of PRIT-Dg, **b** galectin-Dg1, and **c** the interdomain repeats of PRIT-Dg (red boxes). The final two lanes of each blot represent use of the co-IP protocol in the absence of glue (negative controls). Blots **a** and **b** originated from the same membrane which was cut in two in order to allow detection of PRIT-Dg and galectin-Dg1 in parallel, given their size difference (> 180 kDa and ~ 16 kDa, respectively). Sizes of the closest molecular weight markers are indicated in kDa.

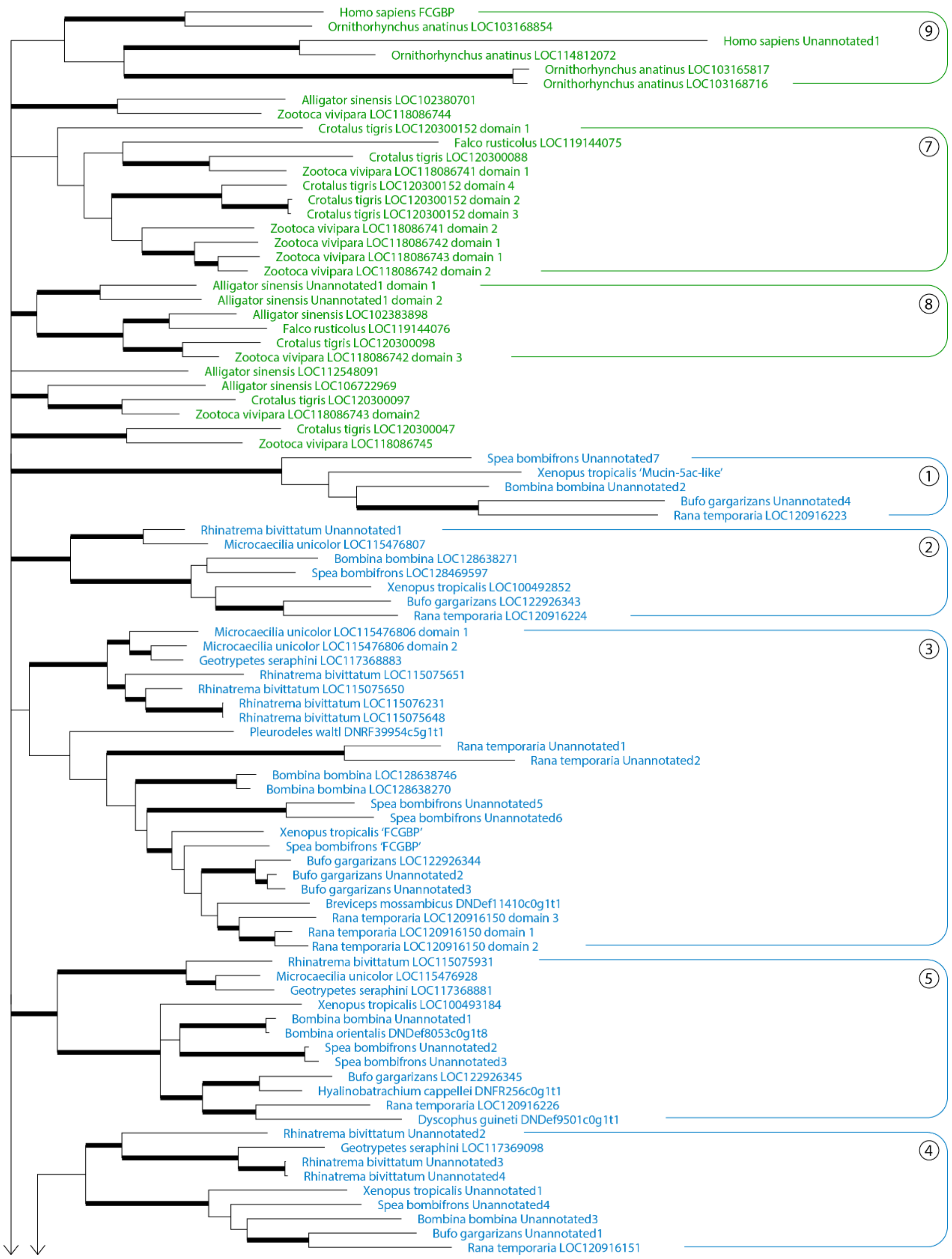

Supplementary Figure 9. Phylogenetic reconstruction of IgGFcBD from 167 proteins containing this domain (Supplementary Data 2). Bold black branches indicate high support (Bayesian posterior probability ≥ 0.95; maximum likelihood bootstrap values ≥ 70%). IgGFcBD from amphibians are marked in blue, whereas those from all other vertebrates are in green. PRIT sequences from glue-producing species are denoted in orange. Clade numbers correspond to associated genes in the evolutionary conserved cluster encoding IgGFcBD-containing proteins (Fig. 3a). Accessions beginning with 'DN' originate from in-house assembled skin transcriptome libraries, while all other accessions were obtained from public databases.

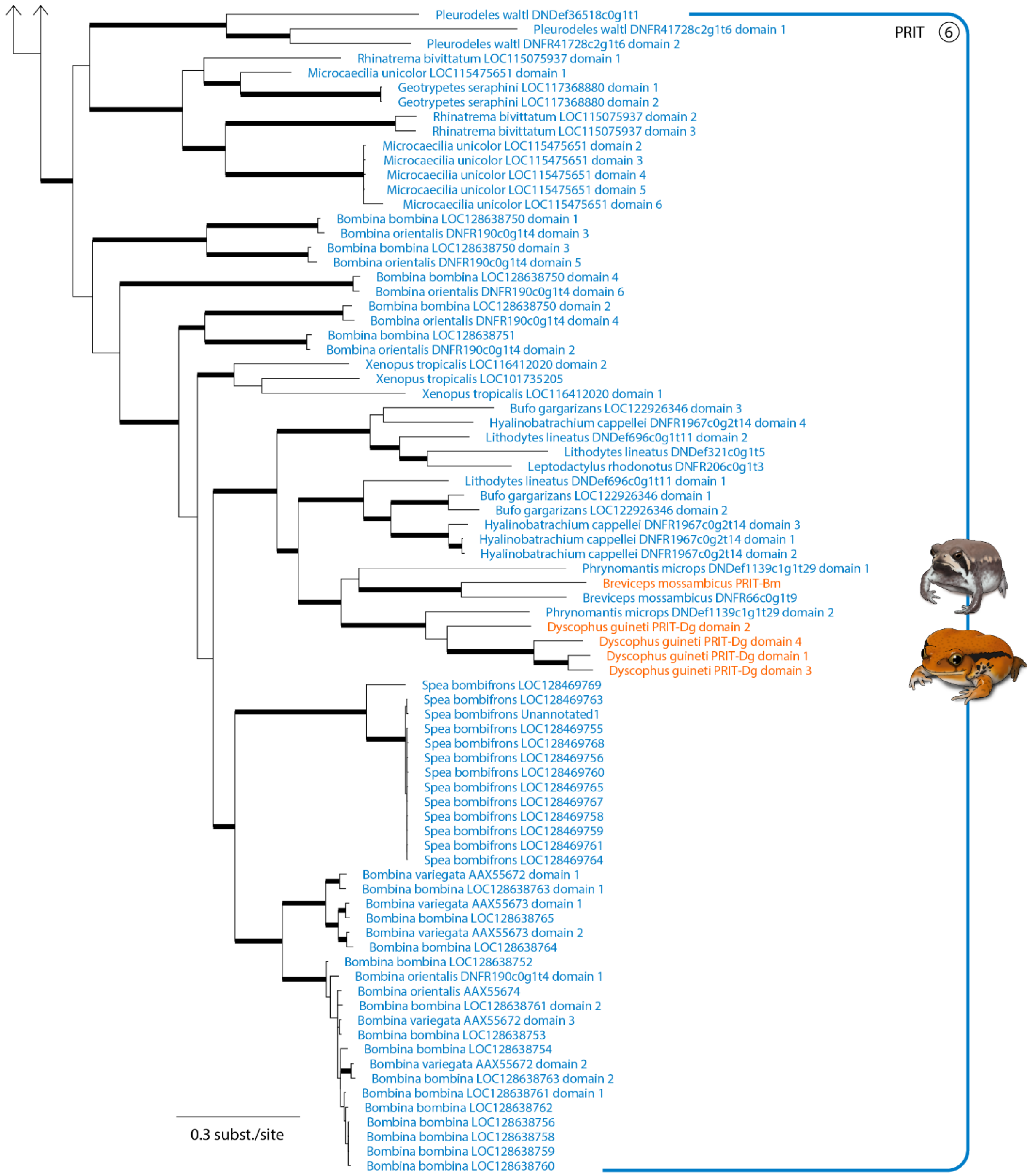

Supplementary Figure 9. (continued)

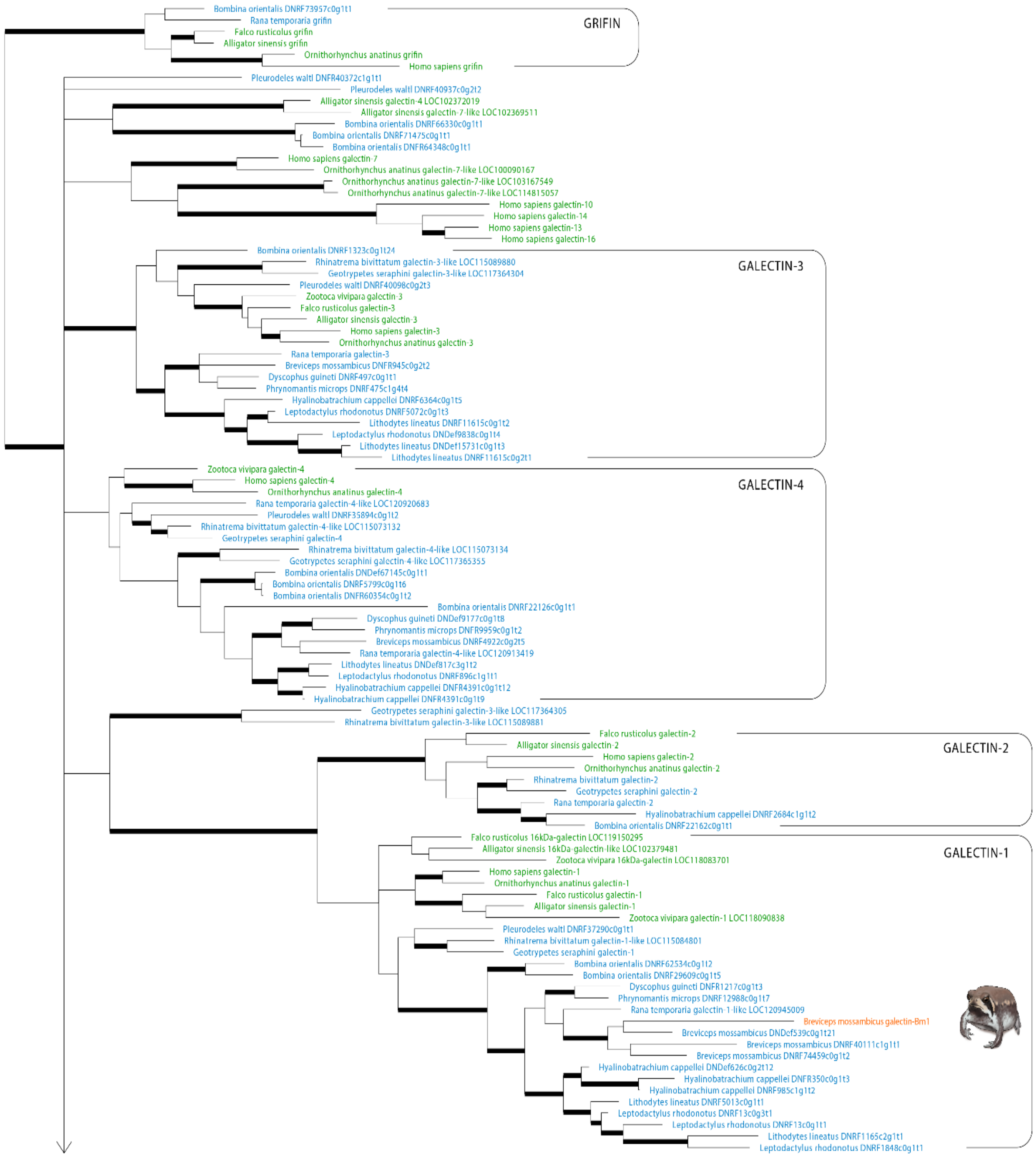

Supplementary Figure 10. Phylogenetic reconstruction of 179 galectin sequences (Supplementary Data 3). Bold black branches indicate high support (Bayesian posterior probability ≥ 0.95; maximum likelihood bootstrap values ≥ 70%). Galectins from amphibians are marked in blue, whereas those from all other vertebrates are in green. The highest expressed galectins in glue-producing frogs are marked in orange. Accessions beginning with 'DN' originate from in-house assembled skin transcriptome libraries, while all other accessions were obtained from public databases.

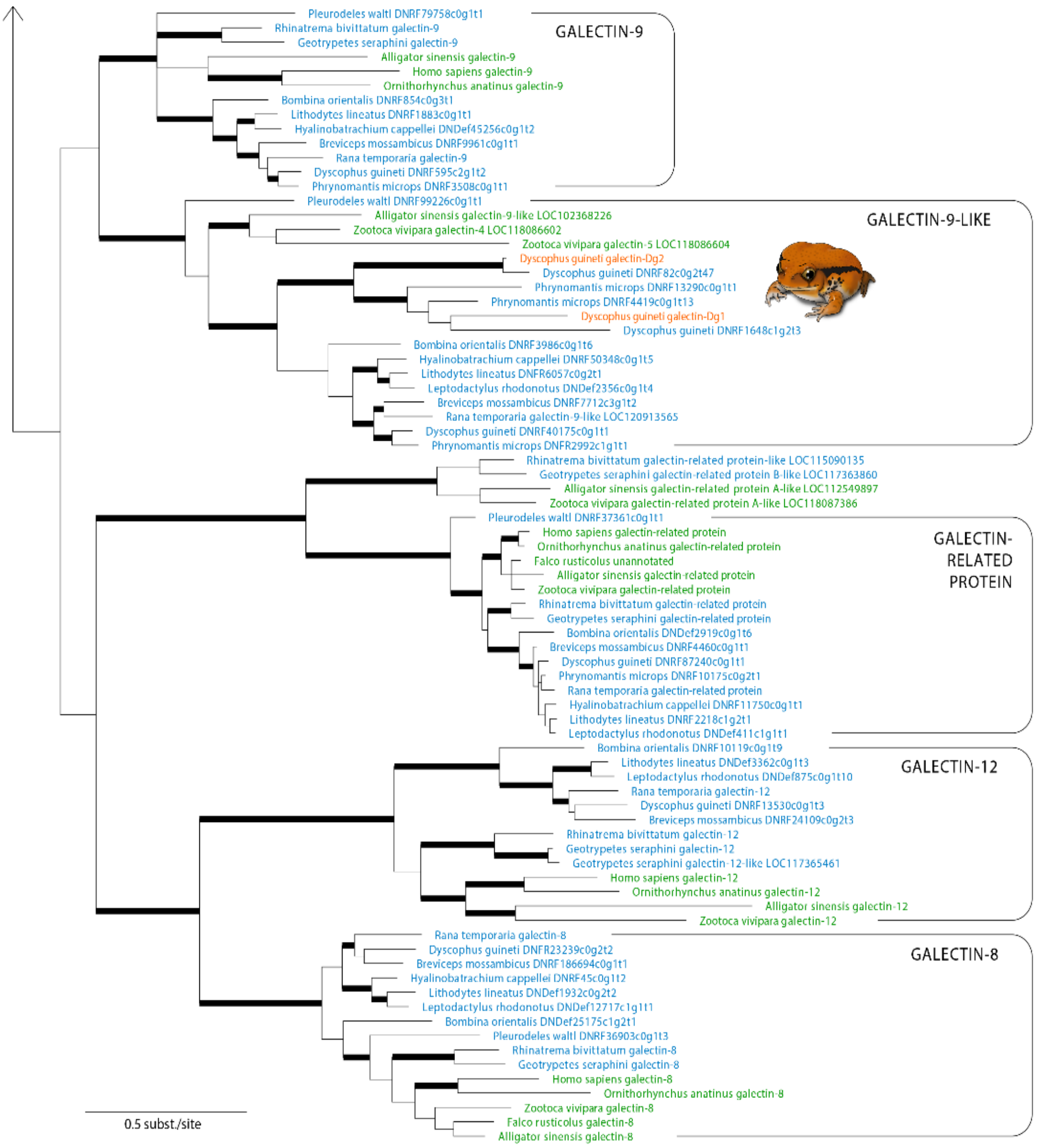

Supplementary Figure 10. (continued)

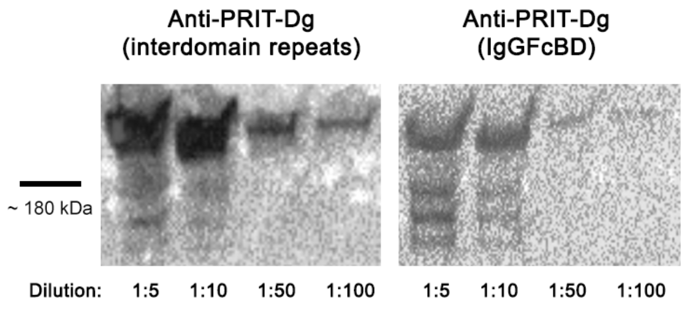

Supplementary Figure 11. Polyclonal antibodies were raised against two peptides within the *D. guineti* glue protein PRIT-Dg1: one located in the interdomain repeat region between two successive IgGFcBD (left) and the other in one such domain itself (right). While both antibodies resulted in identical banding patterns when used in Western blots, the antibody targeting PRIT-Dg1's interdomain repeats consistently achieved higher sensitivity (i.e., generated stronger signals at shorter exposure times). The size of the highest molecular weight marker used is indicated in kDa.
