# Supplementary material for: Recurrent evolution of adhesive defence systems in amphibians by parallel shifts in gene expression
Source: Nat Commun. 2024 Jul 10;15:5612. doi: 10.1038/s41467-024-49917-3 (PMC11237159; doi:10.1038/s41467-024-49917-3)
Supplement: Supplementary file 3 — Reporting Summary [file 41467_2024_49917_MOESM3_ESM.pdf]

nature portfolio

Corresponding author(s): Kim Roelants
Last updated by author(s): 6/04/2024

Reporting Summary

Nature Portfolio wishes to improve the reproducibility of the work that we publish. This form provides structure for consistency and transparency in reporting. For further information on Nature Portfolio policies, see our Editorial Policies and the Editorial Policy Checklist.

Statistics

For all statistical analyses, confirm that the following items are present in the figure legend, table legend, main text, or Methods section.

n/a	Confirmed	
☐	☒	The exact sample size (n) for each experimental group/condition, given as a discrete number and unit of measurement
☒	☐	A statement on whether measurements were taken from distinct samples or whether the same sample was measured repeatedly
☐	☒	The statistical test(s) used AND whether they are one- or two-sided<br>*Only common tests should be described solely by name; describe more complex techniques in the Methods section.*
☒	☐	A description of all covariates tested
☒	☐	A description of any assumptions or corrections, such as tests of normality and adjustment for multiple comparisons
☐	☒	A full description of the statistical parameters including central tendency (e.g. means) or other basic estimates (e.g. regression coefficient) AND variation (e.g. standard deviation) or associated estimates of uncertainty (e.g. confidence intervals)
☐	☒	For null hypothesis testing, the test statistic (e.g. F, t, r) with confidence intervals, effect sizes, degrees of freedom and P value noted<br>*Give P values as exact values whenever suitable.*
☐	☒	For Bayesian analysis, information on the choice of priors and Markov chain Monte Carlo settings
☒	☐	For hierarchical and complex designs, identification of the appropriate level for tests and full reporting of outcomes
☒	☐	Estimates of effect sizes (e.g. Cohen's d, Pearson's r), indicating how they were calculated

Our web collection on statistics for biologists contains articles on many of the points above.

Software and code

Policy information about availability of computer code

Data collection	No software was used for data collection.
Data analysis	For de novo transcriptome assembly and analysis, the following software was used: FastQC 0.11.9, Trinity 2.15.1, EvidentialGene (http://arthropods.eugenes.org/EvidentialGene/trassembly.html), kallisto 0.44, BUSCO 5.4.5, BLAST+ 2.13.0. For screening and identification of glue candidates, the Conserved Domains Database (CDD) of NCBI and InterPro were used. Sanger sequencing data were aligned using CodonCode Aligner 10.0.2 and MAFFT 7.0. Structural predictions were carried out with NetNGlyc 1.0, NetOGlyc 4.0, ProtParam, IUPRed3, GOR 4 and AlphaFold2 2.1.1. Phylogenetic analysis involved the use of MAFFT 7.0, ModelTest-NG v0.1.7, RAxML and MrBayes 3.2.7, while selection analyses were facilitated by TranslatorX and conducted with the pamlX software package. Sequenced peptides and mass spectra were analysed using Xcalibur 3.1.66.10, MASCOT version 2.2.06, Scaffold 3.6.5 and Progenesis version 4.0. Statistical analyses were carried out using R version 4.3.3 (packages lme4 and lsmeans).

For manuscripts utilizing custom algorithms or software that are central to the research but not yet described in published literature, software must be made available to editors and reviewers. We strongly encourage code deposition in a community repository (e.g. GitHub). See the Nature Portfolio guidelines for submitting code & software for further information.

Data

Policy information about availability of data

All manuscripts must include a data availability statement. This statement should provide the following information, where applicable:

- Accession codes, unique identifiers, or web links for publicly available datasets
- A description of any restrictions on data availability
- For clinical datasets or third party data, please ensure that the statement adheres to our policy

Nucleotide and protein sequences have been deposited in GenBank under accession numbers OR483821 (PRIT-Dg), OR480786 (galectin-Dg1), OR480787 (galectin-Dg2), OR480788 (PRIT-Bm) and OR543004 (galectin-Bm1). All other relevant data analysed in this study are available within the Article, Supplementary Information or Source Data file.

Research involving human participants, their data, or biological material

Policy information about studies with human participants or human data. See also policy information about sex, gender (identity/presentation), and sexual orientation and race, ethnicity and racism.

Reporting on sex and gender	The study did not involve human participants, their data, or biological material.
Reporting on race, ethnicity, or other socially relevant groupings	The study did not involve human participants, their data, or biological material.
Population characteristics	The study did not involve human participants, their data, or biological material.
Recruitment	The study did not involve human participants, their data, or biological material.
Ethics oversight	The study did not involve human participants, their data, or biological material.

Note that full information on the approval of the study protocol must also be provided in the manuscript.

Field-specific reporting

Please select the one below that is the best fit for your research. If you are not sure, read the appropriate sections before making your selection.

☐ Life sciences ☐ Behavioural & social sciences ☒ Ecological, evolutionary & environmental sciences

For a reference copy of the document with all sections, see nature.com/documents/nr-reporting-summary-flat.pdf

Ecological, evolutionary & environmental sciences study design

All studies must disclose on these points even when the disclosure is negative.

Study description	This study was designed to investigate the adhesive skin secretion produced by Dyscophus guineti (Microhylidae) to gain insights into the molecular basis, functioning and evolution of this antipredatory defence weapon.
Research sample	The research sample consisted of adult captive-bred Dyscophus guineti frogs (n = 10). This species was selected due to the adhesive nature of its skin secretion as well as its availability in the pet trade, both factors that render it amenable as a model organism for the study of frog glue. Sex and age were not determined as they were not relevant to our objectives.
Sampling strategy	Since the purpose of the experiment was to identify molecules responsible for adhesion (i.e., glue proteins), no statistical analyses were required. Consequently, a small number of animals (n = 7) was sufficient to capture potential individual variation. Instead, the number of required animals was determined by the need to (i) sacrifice individuals for collection of skin tissues (e.g., for RNAseq library construction and immunohistochemistry; n = 3), and (ii) retain multiple living individuals to alternate sampling of secretion in successive rounds (n = 7).
Data collection	Tensile strength measurements were carried out by K.R. and S.Z. using mechanical pull tests and a dynamometer. Transcriptome analyses were carried out by S.Z. and K.R. and involved RNA-seq library assembly and PCR-based methods. Proteomic analyses were carried out by S.C. and S.Z. using tandem mass spectrometry.
Timing and spatial scale	Tissue sampling for transcriptomic analyses and immunohistochemistry experiments were carried out in 2018 and 2021, respectively. Sampling of skin secretions for proteomic analyses was carried out in 2021. For immunolabeling of skin secretions, sampling was carried out in 2019 (for PRIT-Dg) and 2021 (for galectin-Dg1).
Data exclusions	No data were excluded from the analyses.

Reproducibility	All attempts to repeat the experiments were successful.
Randomization	Since our study does not include any comparisons between different treatment groups, randomization was irrelevant.
Blinding	Blinding was either irrelevant or unfeasible due to the nature of our measurements; however, this has no effect on the outcome and conclusions of our study.

Did the study involve field work? ☐ Yes ☒ No

Reporting for specific materials, systems and methods

We require information from authors about some types of materials, experimental systems and methods used in many studies. Here, indicate whether each material, system or method listed is relevant to your study. If you are not sure if a list item applies to your research, read the appropriate section before selecting a response.

Materials & experimental systems

n/a	Involved in the study
☐	☒ Antibodies
☒	☐ Eukaryotic cell lines
☒	☐ Palaeontology and archaeology
☐	☒ Animals and other organisms
☒	☐ Clinical data
☒	☐ Dual use research of concern
☒	☐ Plants

Methods

n/a	Involved in the study
☒	☐ ChIP-seq
☒	☐ Flow cytometry
☒	☐ MRI-based neuroimaging

Antibodies

Antibodies used	Based on the protein sequences described in the study, antigenic peptides were identified, synthesised and used to raise custom polyclonal antibodies against three different targets: (1) an IgGFcBD in PRIT-Dg (QANFKKEMKVRKGQT); (2) the repeat region in between two successive IgGFcBD of PRIT-Dg (QIITEEIPGRPEIPG); and (3) galectin-Dg1 (GPGDNFEVEIRNEG). All of the above steps were outsourced to Eurogentec (Seraing, Belgium).
Validation	Polyclonal antibodies were all subjected to small affinity purification. Antibody activity and analysis of the purified antibodies was determined by indirect ELISA, and compared to samples collected at previous stages of the immunisation program and antibody production process (preimmune serum, large bleed, final bleed; outsourced to Eurogentec). Antibody specificity was verified in-house using Western Blots on D. guineti skin secretion.

Animals and other research organisms

Policy information about studies involving animals; ARRIVE guidelines recommended for reporting animal research, and Sex and Gender in Research

Laboratory animals	The study involved live, captive-bred adult Dyscophus guineti frogs obtained from the commercial pet trade: specifically, the specialist pet stores "Anaconda Reptiles" (Kontich, Belgium) and "Hobbyzoo Anura" (Vlodrop, Netherlands).
Wild animals	The study did not involve wild animals.
Reporting on sex	This information has not been collected due to the irrelevance of sex on the presence of glue, especially within a macroevolutionary framework.
Field-collected samples	The study did not involve samples collected from the field.
Ethics oversight	Experiments involving live frogs were conducted in accordance with European guidelines and Belgian legislation on animal housing and experimentation. All procedures were approved by the Ethical Committee of Animal Experimentation of the Vrije Universiteit Brussel (permit no. EC16-334-1). Animals were euthanised using a 10% lidocaine solution (10 µL/g body weight).

Note that full information on the approval of the study protocol must also be provided in the manuscript.
