# Supplementary material for: Recurrent evolution of adhesive defence systems in amphibians by parallel shifts in gene expression
Source: Nat Commun. 2024 Jul 10;15:5612. doi: 10.1038/s41467-024-49917-3 (PMC11237159; doi:10.1038/s41467-024-49917-3)
Supplement: Supplementary file 4 — Description of Additional Supplementary Files [file 41467_2024_49917_MOESM4_ESM.pdf]

Description of Additional Supplementary Files

File Name: Supplementary Data 1

Description: Expression levels in transcripts per million (tpm) of housekeeping genes and randomly selected single-copy orthologs across eight amphibian skin libraries.

File Name: Supplementary Data 2

Description: Sequences of proteins containing the IgGFc binding domain (IgGFcBD) obtained from amphibian skin transcriptomes and publicly available databases.

File Name: Supplementary Data 3

Description: Sequences of galectin proteins obtained from amphibian skin transcriptomes and publicly available databases.
