# Supplementary material for: Recurrent evolution of adhesive defence systems in amphibians by parallel shifts in gene expression
Source: Nat Commun. 2024 Jul 10;15:5612. doi: 10.1038/s41467-024-49917-3 (PMC11237159; doi:10.1038/s41467-024-49917-3)
Supplement: Supplementary file 5 — Dataset 1 [file 41467_2024_49917_MOESM5_ESM.docx]

|  | ***Dyscophus guineti*** | ***Breviceps mossambicus*** | ***Phrynomantis microps*** | ***Lithodytes lineatus*** | ***Leptodactylus rhodonotus*** | ***Hyalinobatrachium cappellei*** | ***Bombina orientalis*** | ***Pleurodeles waltl*** |
| --- | --- | --- | --- | --- | --- | --- | --- | --- |
| calnexin | 11.65 | 138.01 | 42.41 | 84.87 | 30.45 | 138.83 | 62.33 | 90.94 |
| cytochrome c1 | 14.34 | 90.58 | 47.11 | 48.14 | 62.49 | 47.56 | 28.58 | 83.24 |
| beta-glucurodinase | 7.96 | 3.74 | 14.46 | 10.43 | 12.27 | 4.10 | 5.64 | 4.93 |
| hypoxanthine-guanine phosphoribosyltransferase | 4.99 | 7.87 | 10.73 | 3.23 | 2.57 | 11.55 | 13.48 | 33.12 |
| succinate dehydrogenase complex, subunit A | 18.55 | 49.20 | 47.65 | 90.58 | 38.64 | 28.55 | 26.01 | 56.19 |
| TATA box binding protein | 3.01 | 2.43 | 8.07 | 6.61 | 10.59 | 10.62 | 5.54 | 3.72 |
| Ubiquitin protein ligase E3 component n-recognin 4 | 2.17 | 9.42 | 4.31 | 4.67 | 4.62 | 6.08 | 9.01 | 4.82 |
| O-linked N-acetylglucosamine (GlcNAc) transferase | 29.73 | 33.65 | 47.87 | 90.45 | 143.91 | 58.99 | 51.97 | 32.18 |
| VPS16, CORVET/HOPS core subunit | 7.03 | 4.01 | 11.06 | 6.48 | 3.01 | 8.67 | 8.24 | 5.79 |
| alpha-adducin isoform X1 | 4.76 | 8.36 | 2.47 | 2.82 | 2.21 | 12.79 | 17.92 | 16.50 |
| Alkylglycerone-phosphate synthase | 2.08 | 21.32 | 1.91 | 2.35 | 3.43 | 4.76 | 16.67 | 4.97 |
| CWF19-like protein 1 | 3.35 | 3.35 | 5.50 | 1.85 | 2.71 | 7.83 | 7.43 | 10.40 |
| V-type proton ATPase subunit H | 17.93 | 14.03 | 39.73 | 24.67 | 22.47 | 14.55 | 37.12 | 27.72 |
| heat shock 70 kDa protein 14 | 8.30 | 4.02 | 8.14 | 6.75 | 6.65 | 9.54 | 15.07 | 8.54 |
| polymerase delta-interacting protein 3 | 6.68 | 15.18 | 15.86 | 12.19 | 12.11 | 48.42 | 10.73 | 6.73 |
| diphthamide biosynthesis protein 1 | 1.61 | 4.65 | 2.70 | 3.41 | 3.77 | 4.58 | 4.21 | 5.20 |
| E74 like ETS transcription factor 3 | 2.76 | 102.58 | 48.27 | 10.89 | 4.42 | 3.65 | 228.71 | 23.98 |
| transcriptional adapter 1 | 1.76 | 3.18 | 2.81 | 1.67 | 4.48 | 14.87 | 3.88 | 3.60 |
| Solute carrier family 30 member 7 | 8.21 | 5.42 | 7.97 | 15.77 | 4.72 | 41.56 | 9.49 | 9.74 |
| malonyl-CoA-acyl carrier protein transacylase, mitochondrial | 1.65 | 4.28 | 2.15 | 4.39 | 2.25 | 4.89 | 2.42 | 4.10 |
| zinc finger MYND domain-containing protein 19 | 1.85 | 10.54 | 6.67 | 1.87 | 4.05 | 14.68 | 3.84 | 3.53 |
| voltage-gated hydrogen channel 1 | 12.81 | 8.07 | 30.87 | 13.22 | 14.38 | 14.45 | 19.73 | 3.58 |
| V-type proton ATPase 21 kDa proteolipid subunit | 13.64 | 21.00 | 36.13 | 20.92 | 19.03 | 55.65 | 42.15 | 16.83 |
| LYR motif-containing protein 1 | 3.96 | 4.10 | 4.72 | 6.30 | 5.70 | 2.35 | 11.26 | 7.96 |
| nucleolar protein 12 | 6.60 | 9.04 | 10.50 | 3.94 | 6.66 | 18.28 | 21.61 | 14.11 |
| Glycine C-acetyltransferase | 19.62 | 13.71 | 25.19 | 13.05 | 14.84 | 41.85 | 12.09 | 37.64 |
| Golgi brefeldin A resistant guanine nucleotide exchange factor 1 | 2.83 | 5.22 | 6.07 | 3.93 | 2.22 | 7.78 | 5.14 | 4.80 |
| phosphoinositide 3-kinase regulatory subunit 4 | 1.72 | 6.03 | 9.64 | 3.85 | 16.88 | 10.05 | 3.03 | 3.67 |
| ubiquitin carboxyl-terminal hydrolase 47 isoform X1 | 2.35 | 8.90 | 6.38 | 3.66 | 3.28 | 5.99 | 4.83 | 3.52 |
| isoleucine--tRNA ligase, cytoplasmic | 8.82 | 23.27 | 12.93 | 12.94 | 14.95 | 21.05 | 16.33 | 14.14 |
| cyclin-G-associated kinase | 4.10 | 11.74 | 18.16 | 13.93 | 9.05 | 17.21 | 11.08 | 6.27 |
| symplekin | 5.90 | 9.24 | 5.92 | 3.22 | 3.23 | 17.50 | 8.69 | 9.33 |
| polyribonucleotide nucleotidyltransferase 1, mitochondrial | 1.27 | 11.10 | 8.59 | 2.96 | 2.46 | 5.05 | 4.05 | 4.11 |
| Peptidyl-glycine alpha-amidating monooxygenase | 7.95 | 26.61 | 18.85 | 36.66 | 21.24 | 30.39 | 39.67 | 14.24 |
| pre-mRNA-processing factor 17 | 4.23 | 5.51 | 10.45 | 5.81 | 9.22 | 9.78 | 8.34 | 4.23 |
| conserved oligomeric Golgi complex subunit 2 | 5.22 | 3.76 | 5.45 | 5.50 | 4.14 | 5.89 | 13.48 | 8.86 |
| vitamin K-dependent gamma-carboxylase | 2.74 | 4.86 | 6.16 | 3.19 | 6.59 | 7.48 | 20.68 | 2.31 |
| COP1, E3 ubiquitin ligase | 5.19 | 8.41 | 8.21 | 5.75 | 11.61 | 14.04 | 10.84 | 6.03 |
| beta-catenin-like protein 1 | 6.36 | 14.56 | 15.56 | 8.55 | 13.98 | 21.48 | 10.18 | 6.19 |
| putative ATP-dependent RNA helicase DHX33 | 2.18 | 3.72 | 2.69 | 1.29 | 1.19 | 5.35 | 4.26 | 7.61 |
| serine/threonine-protein kinase RIO2 | 8.01 | 9.38 | 17.50 | 7.31 | 7.31 | 17.20 | 8.76 | 31.32 |
| translation initiation factor eIF-2B subunit epsilon | 6.92 | 2.26 | 11.87 | 9.56 | 9.08 | 11.21 | 8.56 | 7.28 |
| RNA polymerase III subunit C | 2.59 | 3.21 | 5.42 | 3.07 | 1.79 | 3.99 | 4.23 | 4.82 |
| Transmembrane and coiled-coil domains 3 | 1.03 | 3.37 | 5.31 | 2.42 | 5.00 | 3.39 | 3.52 | 2.77 |
| UBX domain-containing protein 7 | 1.47 | 5.59 | 5.54 | 1.85 | 1.31 | 3.88 | 2.72 | 2.69 |
| Ferrochelatase | 2.25 | 3.49 | 8.59 | 2.76 | 1.99 | 2.87 | 6.69 | 24.09 |
| thioredoxin domain-containing protein 11 | 2.77 | 3.70 | 5.41 | 1.54 | 3.52 | 9.76 | 2.79 | 4.46 |
| peptidyl-prolyl cis-trans isomerase-like 4 | 13.69 | 14.21 | 15.78 | 6.38 | 19.47 | 29.56 | 11.34 | 16.99 |
| ATP-dependent RNA helicase DDX51 | 2.94 | 3.92 | 4.45 | 3.70 | 3.14 | 5.10 | 2.43 | 2.41 |
| Integrin alpha FG-GAP repeat containing 1 | 3.81 | 6.24 | 4.19 | 10.39 | 6.30 | 15.12 | 16.28 | 11.51 |
| H/ACA ribonucleoprotein complex subunit 4 | 31.95 | 22.63 | 70.16 | 53.03 | 54.87 | 57.48 | 22.45 | 28.44 |
| ERCC excision repair 8, CSA ubiquitin ligase complex subunit | 1.14 | 2.19 | 3.40 | 4.56 | 4.02 | 4.92 | 2.61 | 1.13 |
| DNA-directed RNA polymerase I subunit RPA1 | 1.10 | 1.84 | 4.97 | 1.96 | 3.71 | 4.98 | 3.15 | 2.92 |
| UTP4, small subunit processome component | 2.24 | 5.73 | 5.91 | 3.50 | 2.76 | 9.68 | 3.19 | 4.58 |
| Coiled-coil domain containing 59 | 7.53 | 11.14 | 6.16 | 1.78 | 7.48 | 7.24 | 10.73 | 8.58 |
| zinc finger SWIM domain-containing protein 7 | 1.17 | 4.80 | 7.13 | 3.66 | 4.84 | 4.30 | 6.43 | 2.50 |
| NHL repeat-containing protein 3 | 2.92 | 4.55 | 2.96 | 10.76 | 20.63 | 25.41 | 6.95 | 2.59 |
| neudesin | 7.68 | 4.57 | 8.35 | 4.56 | 4.67 | 7.09 | 9.74 | 7.99 |
| Solute carrier family 35 member C1 | 1.70 | 10.90 | 4.26 | 5.40 | 4.97 | 18.11 | 16.40 | 14.90 |
| O-6-methylguanine-DNA methyltransferase | 29.89 | 39.22 | 4.65 | 17.30 | 28.14 | 9.22 | 15.79 | 4.15 |
| Galactokinase 2 | 9.59 | 3.49 | 12.58 | 10.31 | 12.02 | 10.89 | 8.76 | 6.82 |
| transmembrane protein 128 | 3.57 | 2.89 | 4.18 | 6.08 | 6.87 | 12.58 | 13.54 | 2.15 |
| autophagy-related protein 101 | 5.19 | 7.91 | 10.43 | 14.12 | 9.34 | 11.15 | 11.44 | 12.60 |
| kelch-like protein 12 | 1.01 | 3.10 | 13.57 | 3.75 | 3.17 | 3.87 | 3.88 | 1.84 |
| putative GTP-binding protein 6 | 2.10 | 3.54 | 5.67 | 2.77 | 3.35 | 5.18 | 4.09 | 2.82 |
| TRAF family member-associated NF-kappa-B activator isoform X1 | 2.32 | 18.64 | 9.97 | 22.37 | 9.54 | 10.26 | 13.41 | 8.28 |
| WD repeat-containing protein 70 | 3.25 | 28.14 | 9.74 | 2.15 | 4.37 | 5.24 | 15.32 | 7.13 |
| TBC1 domain family member 30 | 4.04 | 4.56 | 6.09 | 21.44 | 4.03 | 5.46 | 9.79 | 1.64 |
| maspardin | 7.80 | 15.19 | 15.13 | 5.00 | 3.93 | 7.42 | 13.11 | 6.93 |
| 60S ribosome subunit biogenesis protein NIP7 homolog | 13.14 | 25.37 | 31.46 | 15.63 | 13.00 | 20.97 | 17.72 | 15.10 |
| tRNA-yW synthesizing protein 5 | 1.27 | 1.38 | 6.26 | 5.01 | 8.41 | 12.71 | 3.10 | 3.73 |
| myeloid differentiation primary response protein MyD88 | 3.29 | 4.46 | 15.19 | 13.19 | 2.83 | 19.54 | 20.38 | 12.42 |
| Coiled-coil domain containing 32 | 6.67 | 3.58 | 18.11 | 18.48 | 11.70 | 7.00 | 6.85 | 3.92 |
| COMM domain-containing protein 2 | 6.08 | 8.26 | 24.46 | 15.35 | 10.62 | 26.90 | 14.97 | 3.32 |
| trafficking protein particle complex subunit 11 | 2.99 | 7.35 | 7.30 | 5.42 | 4.68 | 10.06 | 6.08 | 5.07 |
| Iron-sulfur cluster assembly 2 | 28.91 | 9.94 | 16.73 | 17.33 | 11.75 | 0.30 | 9.46 | 6.53 |
| Basic helix-loop-helix family member a15 | 5.04 | 5.51 | 6.16 | 10.87 | 8.46 | 3.76 | 10.09 | 2.17 |
| Nucleoporin 153 | 2.37 | 7.33 | 6.80 | 1.75 | 3.45 | 5.58 | 7.12 | 4.36 |
| 26S proteasome non-ATPase regulatory subunit 2 | 21.01 | 41.31 | 58.13 | 43.90 | 30.87 | 65.32 | 29.13 | 52.99 |
| protein Dr1 | 0.93 | 2.03 | 4.20 | 1.82 | 1.77 | 3.01 | 4.91 | 6.02 |
| serine protease 23 | 5.21 | 3.08 | 14.94 | 6.17 | 7.79 | 1.75 | 18.18 | 7.79 |
| PRKCA-binding protein | 7.88 | 7.11 | 19.12 | 15.16 | 8.41 | 5.82 | 10.65 | 6.11 |
| DNA-directed RNA polymerase III subunit RPC5 | 8.53 | 6.50 | 8.67 | 4.83 | 12.56 | 5.08 | 5.63 | 1.65 |
| ubiquitin carboxyl-terminal hydrolase 14 | 10.89 | 25.49 | 31.91 | 14.95 | 10.61 | 25.80 | 23.31 | 17.44 |
| transmembrane protein 248 | 19.26 | 29.07 | 16.07 | 21.71 | 128.70 | 41.03 | 46.94 | 9.24 |
| Transmembrane protein 39B | 4.33 | 5.28 | 8.07 | 8.37 | 16.82 | 17.21 | 8.78 | 4.34 |
| exosome complex component RRP40 | 4.76 | 9.94 | 14.04 | 6.68 | 11.55 | 21.46 | 9.12 | 13.17 |
| cap-specific mRNA (nucleoside-2'-O-)-methyltransferase 1 | 4.66 | 7.82 | 13.90 | 2.51 | 3.23 | 9.01 | 3.34 | 7.04 |
| nudC domain-containing protein 2 | 4.85 | 5.78 | 6.20 | 14.06 | 6.10 | 26.87 | 19.15 | 9.76 |
| RNA transcription, translation and transport factor | 16.74 | 102.48 | 53.16 | 60.47 | 59.97 | 63.31 | 52.97 | 65.10 |
| tRNA (adenine(58)-N(1))-methyltransferase non-catalytic subunit TRM6 | 4.93 | 8.30 | 8.45 | 5.14 | 4.98 | 10.45 | 6.41 | 5.60 |
| protein RER1 | 11.19 | 54.91 | 37.88 | 40.20 | 15.86 | 66.79 | 67.33 | 25.98 |
| probable tRNA pseudouridine synthase 2 | 10.22 | 11.64 | 10.36 | 7.42 | 5.58 | 18.18 | 4.07 | 4.42 |
| SAP30-binding protein | 18.44 | 3.31 | 18.62 | 7.33 | 13.25 | 11.11 | 5.46 | 10.38 |
| protein Red | 14.87 | 30.54 | 25.30 | 24.88 | 20.78 | 39.64 | 22.33 | 16.69 |
| endothelial cell-specific molecule 1 | 1.15 | 1.18 | 0.98 | 1.33 | 6.16 | 1.51 | 11.17 | 2.14 |
| RWD domain-containing protein 4 | 41.82 | 23.90 | 28.99 | 23.86 | 24.53 | 23.81 | 20.98 | 10.57 |
| UPF0505 protein C16orf62 homolog | 3.64 | 2.81 | 5.03 | 2.16 | 2.78 | 6.12 | 5.17 | 3.24 |
| zinc finger CCHC domain-containing protein 8 | 2.83 | 4.75 | 17.10 | 5.44 | 8.08 | 11.29 | 10.73 | 5.60 |
| Alpha tocopherol transfer protein like | 2.57 | 8.47 | 9.00 | 1.68 | 1.54 | 4.75 | 16.47 | 2.77 |
| protein FAM134A | 2.97 | 3.33 | 12.66 | 6.23 | 3.11 | 7.46 | 14.02 | 1.51 |
| PHD finger protein 13 | 1.81 | 2.54 | 4.79 | 6.06 | 1.81 | 3.00 | 2.66 | 2.02 |
| F-box only protein 7 | 1.88 | 24.58 | 4.89 | 1.98 | 8.28 | 5.36 | 7.50 | 11.29 |
| protein CNPPD1 | 4.46 | 10.35 | 12.26 | 8.39 | 11.53 | 19.00 | 16.71 | 9.47 |
| BUD13 homolog | 2.26 | 5.64 | 6.08 | 2.49 | 3.18 | 5.90 | 2.93 | 2.22 |
| putative methyltransferase C9orf114 homolog | 2.79 | 2.77 | 5.83 | 4.12 | 2.86 | 7.36 | 5.98 | 3.36 |
| pentatricopeptide repeat-containing protein 1, mitochondrial | 5.57 | 10.14 | 10.61 | 4.13 | 7.75 | 5.33 | 2.10 | 4.36 |
| protein salvador homolog 1 | 4.20 | 6.57 | 10.59 | 7.62 | 7.25 | 11.60 | 9.32 | 12.95 |
| cyclin-H | 4.57 | 5.77 | 9.73 | 7.99 | 10.06 | 11.38 | 6.79 | 15.96 |
| kaptin | 2.00 | 6.86 | 23.82 | 3.61 | 8.25 | 8.19 | 5.84 | 3.56 |
| DNA polymerase epsilon subunit 3 | 6.18 | 15.50 | 25.23 | 18.71 | 12.51 | 25.79 | 4.21 | 11.07 |
| WD repeat-containing protein 18 | 3.92 | 5.02 | 6.78 | 4.85 | 4.71 | 13.12 | 7.52 | 10.11 |
| Coronin | 1.81 | 5.46 | 4.23 | 5.39 | 5.76 | 17.05 | 8.35 | 7.47 |
| transmembrane protein 186 | 1.68 | 4.97 | 2.41 | 2.47 | 3.66 | 2.23 | 2.36 | 3.71 |
| exosome component 10 | 2.77 | 9.13 | 7.10 | 6.68 | 5.12 | 17.87 | 12.98 | 7.62 |
| nucleolar protein 58 | 12.38 | 18.78 | 21.57 | 31.69 | 16.17 | 29.51 | 26.80 | 19.56 |
| coiled-coil domain-containing protein 93 | 15.35 | 16.31 | 15.49 | 13.43 | 24.69 | 12.93 | 9.54 | 5.23 |
| single Ig IL-1-related receptor | 1.36 | 8.99 | 7.21 | 5.71 | 5.68 | 14.41 | 7.91 | 3.62 |
| oral cancer-overexpressed protein 1 | 7.01 | 5.87 | 21.71 | 9.74 | 3.73 | 16.34 | 6.50 | 5.18 |
| speckle targeted PIP5K1A-regulated poly(A) polymerase | 4.15 | 3.70 | 3.74 | 2.29 | 1.41 | 4.27 | 1.86 | 2.16 |
| golgin subfamily A member 5 | 3.18 | 7.47 | 7.56 | 12.18 | 11.72 | 14.24 | 8.46 | 5.15 |
| Abhydrolase domain containing 18 | 3.63 | 2.31 | 11.29 | 3.96 | 13.86 | 3.45 | 3.03 | 4.61 |
| elongator complex protein 2 | 2.00 | 3.33 | 5.17 | 1.82 | 1.95 | 4.00 | 3.61 | 5.51 |
| Leucine rich repeat containing 28 | 7.01 | 19.48 | 2.22 | 13.01 | 11.73 | 20.48 | 22.75 | 5.83 |
| zinc finger protein-like 1 | 4.10 | 9.95 | 11.16 | 7.57 | 4.69 | 13.83 | 10.30 | 6.90 |
| UV-stimulated scaffold protein A | 1.03 | 4.12 | 7.84 | 4.99 | 8.50 | 10.59 | 3.75 | 2.56 |
| polyphosphoinositide phosphatase | 3.63 | 1.47 | 2.85 | 3.74 | 4.13 | 8.90 | 7.25 | 3.47 |
| charged multivesicular body protein 7 | 2.76 | 5.58 | 11.82 | 3.17 | 2.42 | 4.48 | 11.12 | 3.48 |
| metaxin-2 | 11.33 | 8.98 | 18.96 | 24.52 | 23.47 | 31.12 | 8.82 | 21.10 |
| TPX2, microtubule nucleation factor | 1.81 | 3.69 | 9.89 | 3.02 | 3.73 | 9.64 | 6.71 | 1.94 |
| tRNA-splicing endonuclease subunit Sen2 | 8.05 | 4.25 | 13.79 | 4.52 | 4.81 | 5.68 | 5.02 | 3.94 |
| Eukaryotic translation initiation factor 2B subunit gamma | 3.92 | 20.34 | 7.91 | 7.04 | 13.90 | 12.79 | 8.63 | 17.73 |
| Oxidoreductase NAD binding domain containing 1 | 3.35 | 11.32 | 10.07 | 5.25 | 2.66 | 6.29 | 7.93 | 13.74 |
| Fasciculation and elongation protein zeta 1 | 2.07 | 16.73 | 8.21 | 4.77 | 9.79 | 24.77 | 6.60 | 4.75 |
| nucleoporin Nup43 | 5.80 | 5.31 | 8.98 | 5.33 | 3.65 | 8.12 | 6.51 | 4.05 |
| manganese-transporting ATPase 13A1 | 4.73 | 11.00 | 9.14 | 6.92 | 7.41 | 14.99 | 9.21 | 5.82 |
| dolichyl-diphosphooligosaccharide--protein glycosyltransferase subunit 2 isoform X1 | 37.04 | 86.00 | 55.36 | 106.39 | 52.88 | 100.13 | 53.25 | 120.38 |
| GINS complex subunit 1 | 4.06 | 2.10 | 2.81 | 2.34 | 3.11 | 4.28 | 4.04 | 8.45 |
| ubiquitin-protein ligase E3B | 1.31 | 1.64 | 4.35 | 3.07 | 4.13 | 8.07 | 2.59 | 3.19 |
| negative elongation factor C/D | 6.63 | 5.85 | 13.09 | 8.05 | 7.12 | 10.41 | 5.99 | 4.48 |
| cleavage stimulation factor subunit 3 | 2.98 | 15.05 | 7.65 | 3.08 | 3.42 | 4.86 | 3.83 | 8.55 |
| protein KTI12 homolog | 4.17 | 20.87 | 12.89 | 4.66 | 8.55 | 12.32 | 13.43 | 13.68 |
| oxidoreductase HTATIP2 | 6.18 | 14.39 | 25.05 | 10.12 | 39.78 | 121.18 | 18.49 | 25.39 |
| ER membrane protein complex subunit 2 | 7.24 | 36.43 | 21.95 | 29.62 | 23.60 | 23.81 | 30.73 | 19.65 |
| acyl-coenzyme A thioesterase 13 | 6.64 | 6.85 | 67.87 | 14.69 | 4.70 | 21.33 | 6.53 | 15.62 |
| uncharacterized protein C1orf131 homolog | 11.62 | 18.83 | 15.12 | 4.26 | 4.80 | 11.09 | 6.64 | 6.62 |
| LAS1 like, ribosome biogenesis factor | 2.09 | 6.53 | 8.19 | 7.15 | 4.37 | 7.77 | 5.71 | 8.75 |
| yae1 domain-containing protein 1 | 5.82 | 3.25 | 8.89 | 7.87 | 4.31 | 8.29 | 5.91 | 3.38 |
| alpha-1,3/1,6-mannosyltransferase ALG2 | 5.80 | 2.48 | 6.43 | 12.30 | 12.45 | 16.91 | 5.41 | 18.41 |
| dehydrogenase/reductase SDR family member 7B | 2.03 | 5.74 | 12.11 | 10.01 | 9.04 | 12.71 | 7.80 | 7.94 |
| methylosome protein 50 | 10.93 | 11.87 | 13.96 | 13.44 | 10.62 | 23.79 | 5.01 | 8.74 |
| Mitochondrial ribosomal protein S7 | 6.22 | 9.52 | 16.50 | 11.46 | 10.32 | 9.22 | 8.35 | 7.34 |
| Molybdopterin synthase catalytic subunit | 6.49 | 14.39 | 24.06 | 4.40 | 8.12 | 7.91 | 24.82 | 14.51 |
| Embryonic ectoderm development | 2.84 | 9.93 | 7.95 | 6.45 | 11.54 | 11.54 | 8.63 | 4.84 |
| protein MON2 homolog isoform X1 | 5.94 | 5.43 | 7.26 | 8.04 | 9.47 | 9.07 | 6.39 | 4.06 |
| protein YIPF6 | 16.96 | 9.15 | 13.25 | 19.93 | 26.37 | 31.13 | 18.21 | 13.80 |
| CWF19-like protein 2 | 3.15 | 2.28 | 4.15 | 1.80 | 3.34 | 5.78 | 2.95 | 2.01 |
| nuclear pore complex protein Nup85 | 6.96 | 13.37 | 10.87 | 7.79 | 7.26 | 10.64 | 5.33 | 6.06 |
| Methyltransferase like 18 | 2.03 | 4.02 | 3.87 | 4.85 | 4.75 | 4.05 | 3.18 | 2.48 |
| Ribokinase | 1.68 | 6.20 | 2.82 | 7.18 | 4.00 | 12.44 | 4.78 | 7.63 |
| reticulocalbin-2 | 7.52 | 14.96 | 16.18 | 9.34 | 8.70 | 6.44 | 3.56 | 5.36 |
| Beta-1,4-galactosyltransferase 7 | 3.99 | 2.83 | 3.35 | 3.86 | 9.01 | 4.76 | 3.57 | 2.91 |
| F-box/LRR-repeat protein 15 | 1.43 | 2.80 | 3.30 | 2.47 | 2.44 | 3.85 | 1.80 | 2.48 |
| biogenesis of lysosome-related organelles complex 1 subunit 5 | 10.24 | 3.47 | 24.39 | 3.58 | 7.95 | 19.69 | 5.68 | 4.91 |
| WW domain-containing oxidoreductase | 1.16 | 1.15 | 4.11 | 1.96 | 2.31 | 5.11 | 3.51 | 3.63 |
| transforming growth factor-beta receptor-associated protein 1 | 2.72 | 6.24 | 5.49 | 4.14 | 5.10 | 4.46 | 2.79 | 1.43 |
| actin-related protein 10 | 7.63 | 24.42 | 18.91 | 9.65 | 19.37 | 23.52 | 10.39 | 17.52 |
| pigment epithelium-derived factor | 9.63 | 17.26 | 16.06 | 6.02 | 28.31 | 6.90 | 25.40 | 20.41 |
| importin-9 | 4.42 | 2.77 | 6.72 | 3.41 | 3.70 | 6.39 | 4.98 | 5.20 |
| lysophosphatidic acid receptor 3 | 1.31 | 8.97 | 17.53 | 3.46 | 2.85 | 7.43 | 9.14 | 8.99 |
| leucine-rich repeat-containing protein 57 | 10.68 | 7.25 | 8.59 | 5.74 | 5.07 | 11.29 | 11.04 | 2.18 |
| Glutamyl-tRNA(Gln) amidotransferase subunit B, mitochondrial | 1.64 | 4.11 | 3.98 | 3.10 | 3.97 | 3.49 | 3.98 | 2.21 |
| STAGA complex 65 subunit gamma | 1.39 | 10.27 | 6.08 | 3.00 | 3.72 | 14.68 | 8.12 | 4.03 |
| Replication factor C subunit 1 | 5.37 | 7.35 | 10.05 | 4.39 | 9.09 | 31.43 | 8.89 | 4.18 |
| glucosamine 6-phosphate N-acetyltransferase | 1.72 | 8.31 | 10.33 | 15.33 | 5.35 | 14.19 | 6.22 | 18.55 |
| zinc finger protein ZPR1 | 2.56 | 5.39 | 20.62 | 3.08 | 2.69 | 7.40 | 20.18 | 8.00 |
| DNA primase small subunit | 1.09 | 4.78 | 6.48 | 2.13 | 4.27 | 6.57 | 8.04 | 3.50 |
| Phosphatidate cytidylyltransferase, mitochondrial | 5.66 | 1.86 | 9.22 | 3.32 | 2.48 | 27.76 | 3.38 | 2.82 |
| ankyrin repeat and SOCS box protein 1 | 7.75 | 2.70 | 9.89 | 2.25 | 2.46 | 2.62 | 4.13 | 3.02 |
| synaptopodin | 1.41 | 4.73 | 8.20 | 4.08 | 3.43 | 1.02 | 3.48 | 7.07 |
| transcriptional adapter 2-alpha | 2.33 | 11.87 | 4.94 | 4.59 | 3.43 | 4.91 | 3.22 | 2.65 |
| Centromere protein K | 2.35 | 2.22 | 4.02 | 3.58 | 2.85 | 4.49 | 3.23 | 4.00 |
| transmembrane protein 106C | 3.20 | 17.46 | 7.23 | 9.17 | 3.42 | 10.83 | 12.19 | 19.05 |
| alpha-catulin | 3.67 | 0.67 | 1.51 | 1.67 | 1.94 | 1.26 | 2.58 | 1.68 |
| transcription factor 25 | 4.00 | 10.03 | 7.05 | 13.86 | 13.19 | 24.43 | 7.09 | 26.25 |
| Mitogen-activated protein kinase kinase 7 | 6.34 | 22.85 | 17.21 | 7.89 | 9.20 | 21.11 | 8.56 | 15.35 |
| Zinc finger AN1-type containing 1 | 2.28 | 6.09 | 4.83 | 4.40 | 4.01 | 6.85 | 5.79 | 1.54 |
| KRR1 small subunit processome component homolog | 4.07 | 5.49 | 17.26 | 5.36 | 5.98 | 6.65 | 14.62 | 7.39 |
| protein TFG isoform X1 | 16.57 | 40.06 | 53.80 | 27.67 | 50.79 | 47.66 | 47.20 | 72.62 |
| Matrix metallopeptidase 28 | 2.68 | 2.61 | 21.16 | 4.50 | 6.19 | 13.32 | 7.60 | 1.65 |
| ATP synthase mitochondrial F1 complex assembly factor 2 | 5.11 | 5.94 | 8.56 | 8.25 | 10.43 | 15.71 | 4.92 | 8.41 |
| GPI mannosyltransferase 1 | 4.56 | 5.24 | 8.80 | 2.62 | 1.05 | 3.57 | 4.71 | 3.46 |
| RNA 3'-terminal phosphate cyclase-like protein | 50.57 | 14.09 | 45.95 | 18.90 | 21.00 | 52.27 | 11.78 | 23.40 |
| 26S proteasome non-ATPase regulatory subunit 14 | 9.89 | 27.90 | 32.47 | 18.40 | 14.08 | 46.46 | 52.68 | 20.33 |
| lysine-specific histone demethylase 1B | 3.87 | 5.14 | 6.84 | 3.21 | 4.26 | 8.00 | 6.00 | 1.39 |
| polyribonucleotide 5'-hydroxyl-kinase Clp1 | 1.17 | 6.12 | 3.97 | 5.84 | 4.48 | 11.32 | 3.18 | 5.53 |
| Staufen double-stranded RNA binding protein 1 | 3.40 | 19.60 | 7.04 | 8.14 | 3.62 | 28.66 | 43.21 | 18.87 |
| sin3 histone deacetylase corepressor complex component SDS3 | 3.07 | 3.58 | 1.49 | 5.18 | 5.39 | 4.98 | 4.71 | 6.81 |
| THO complex subunit 3 | 5.55 | 13.58 | 9.12 | 4.12 | 13.07 | 16.87 | 12.29 | 9.59 |
| neuroserpin | 35.26 | 3.60 | 2.38 | 2.57 | 6.32 | 8.69 | 4.29 | 71.81 |
